# Clinical and genetic profiles of postnatal patients with skeletal dysplasia in Guangxi during 8 years: a single-center experience

**DOI:** 10.1186/s13023-026-04414-2

**Published:** 2026-08-31

**Authors:** Sheng Yi, Qi Yang, Linlin Wang, Xunzhao Zhou, Shujie Zhang, Jiale Qian, Shang Yi, Jing Huang, Junjie Chen, Qiang Zhang, Fei Chen, Jiao Li, Shengkai Wei, Xiaofei Zhang, Qinle Zhang, Pingshan Pan, Zailong Qin, Jingsi Luo

**Affiliations:** 1 Genetic and Metabolic Central Laboratory, Guangxi Birth Defects Research and Prevention Institute, Maternal and Child Health Hospital of Guangxi Zhuang Autonomous Region, Nanning, China; 2 Guangxi Clinical Research Center for Birth Defects, Guangxi Clinical Research Center for Pediatric Diseases, Guangxi Key Laboratory of Reproductive Health and Birth Defects Prevention, Guangxi Key Laboratory of Precision Medicine for Genetic Diseases, Guangxi Key Laboratory of Birth Defects and Stem Cell Biobank, Guangxi Key Laboratory of Birth Defects Research and Prevention, Maternal and Child Health Hospital of Guangxi Zhuang Autonomous Region, Nanning, China; 3 Department of Obstetrics, Maternal and Child Health Hospital of Guangxi Zhuang Autonomous Region, Nanning, China; 4 Pediatrics Department, Maternal and Child Health Hospital of Guangxi Zhuang Autonomous Region, Nanning, China; 5 Department of Radiology, Maternal and Child Health Hospital of Guangxi Zhuang Autonomous Region, Nanning, China

**Keywords:** Skeletal dysplasia, Whole-exome sequencing, Molecular diagnosis, Genetic profiles, Clinical experience

## Abstract

**Background:**

Skeletal dysplasia (SD) is a clinically and genetically heterogeneous group of bone and cartilage disorders. Given the typically heterogeneous and nonspecific clinical manifestations associated with these conditions, molecular testing is essential to achieve a definitive diagnosis. To elucidate the clinical and mo^l^ecular characteristics of genetic skeletal disorders in the Guangxi region, we conducted a retrospective single-center study involving 434 families suspected of having SD who underwent molecular analysis in our department over an eight-year period. We analyzed their clinical and molecular genetic data.

**Results:**

Among our cohort, facial dysmorphism and short stature emerged as the most prevalent clinical manifestations, followed by growth delay and motor delay. Notably, molecular diagnoses were made at a relatively late age. Our findings revealed a total of 34 chromosomal abnormalities and 265 Mendelian genetic disorders. According to the Nosology of genetic skeletal disorders: 2023 revision, 75 genes were implicated in 199 positive molecular diagnoses, while the remaining 66 positive diagnoses were attributed to 56 causal genes. The most frequently identified causal gene was FGFR3, with the p.G380R substitution being the predominant pathogenic variant. The overall diagnostic yield through whole-exome sequencing was found to be 64.9%. Predictors of a positive molecular finding included short stature, calvaria abnormalities and knee deformities. Furthermore, the molecular diagnostic rate achieved through Sanger sequencing slightly exceeded that obtained via whole-exome sequencing.

**Conclusions:**

These findings have significantly enhanced the understanding of the clinical and genetic profiles of SD in Guangxi, elucidating the mutation spectrum of the associated causative genes. This study provides valuable clinical experience that can facilitate the effective implementation of genetic testing in the context of SD.

**Clinical trial number:**

Not applicable

**Supplementary Information:**

The online version contains supplementary material available at https://doi.org/10.1186/s13023-026-04414-2.

## Background

Skeletal dysplasia (SD) is a diverse group of genetic disorders that affect the development and architecture of bone and/or cartilage. The clinical manifestations associated with these conditions include a range of abnormalities in the growth and development of skeletal tissue. These may manifest as short stature, craniofacial and limb deformities, abnormal spinal curvature, joint dislocations, and changes in bone mineral density, sometimes combined with other systemic malformations [1–3]. The estimated prevalence of SD is approximately two to three cases per 10,000 births, and these disorders contribute to approximately 2% of perinatal mortality [3–5].

Patients with SD typically exhibit skeletal malformations that affect various anatomical regions and systems within the body, presenting a wide range of clinical phenotypes ranging from mild symptoms to severe consequences, including spontaneous abortion. Many of these diseases exhibit severe symptoms and rapid progression during developmental stages, often accompanied by dysfunction in other organ systems [6]. Consequently, early diagnosis and prompt intervention are crucial for improving the prognosis of individuals affected by SD. Genetic skeletal disorders exhibit considerable clinical heterogeneity and present with non-specific clinical manifestations. This variability poses considerable challenges for clinicians in achieving accurate diagnoses based solely on clinical assessments and radiological imaging. Therefore, a comprehensive understanding of the molecular genetic background of these disorders is essential for effective genetic counseling, the implementation of targeted treatment strategies, and the facilitation of prenatal diagnosis.

The rapid advancement of molecular diagnostic technologies, especially the extensive utilization of high-throughput sequencing, has significantly facilitated the identification of numerous causal genes and variants associated with SD. The 2023 updated edition of the Nosology of genetic skeletal disorders categorizes a total of 771 entries and 552 distinct causative genes into 41 groups according to their clinical, radiographic, and/or molecular characteristics [7]. Nevertheless, the genetic etiology and pathogenic mechanisms underlying certain diseases remain inadequately characterized, and the specific genes and rare variant alleles contributing to SD in patient cohorts have yet to be comprehensively elucidated [7]. Consequently, further investigations are imperative to clarify the molecular mechanisms and phenotypic heterogeneity associated with SD. In this context, we performed a retrospective single-center study over an eight-year period, from July 2015 to December 2023, involving 434 families with suspected SD who underwent targeted Sanger sequencing or whole-exome sequencing (WES) analysis at our department in Guangxi, China. Comprehensive analyses of their clinical presentations and molecular genetic data were systematically conducted.

## Materials and methods

### Patient recruitment

Over an eight-year period, spanning from July 2015 to December 2023, patients exhibiting one or more characteristics of SD- including bone deformities or morphological abnormalities, restricted joint mobility, recurrent fragility fractures, disproportionate short or tall stature, abnormal serum calcium levels, and other related conditions- were consecutively recruited from the Maternal and Child Health Hospital of the Guangxi Zhuang Autonomous Region. This study was conducted in accordance with the ethical guidelines outlined in the Declaration of Helsinki and received approval from the Ethics Committee of the Maternal and Child Health Hospital of the Guangxi Zhuang Autonomous Region (Approval No. G2020-3–11). Informed consent was obtained from each participant or their legal guardians prior to participation.

### WES analysis

Genomic DNA was extracted from peripheral blood lymphocytes using a Lab-Aid DNA kit (Zeesan, China) according to established extraction protocols. Sequencing libraries were generated using an Agilent SureSelect Human All Exon v6 kit (Agilent, CA). The libraries were subsequently pooled, and exome sequencing was performed on a HiSeq2500 platform (Illumina, CA). After removing redundant reads, the sequencing data were aligned to the human reference sequence hg19 using the Burrows-Wheeler Aligner (BWA) and the Genome Analysis Toolkit (GATK HaplotypeCaller; McKenna et al., 2010). Copy number variant (CNV) analysis, based on the read depth method, was performed using an in-house pipeline, and CNVs of interest were further visually inspected with the Integrative Genomics Viewer. Single nucleotide variants and insertions/deletions (indels) were annotated and prioritized by the TGex software (LifeMap Sciences, Alameda, CA). For proband-only WES, all potentially pathogenic variants were validated by Sanger sequencing, while microdeletions or microduplications were verified by multiplex ligation-dependent probe amplification (MLPA) or suitable Gap-PCR in both patients and their parents. The identified variants were evaluated and classified in accordance with American College of Medical Genetics and Genomics (ACMG) and the Association for Molecular Pathology (AMP) guidelines as pathogenic (P), likely pathogenic (LP), or variant of uncertain significance (VUS) [8].

### Sanger sequencing

Each polymerase chain reaction (PCR) was conducted in a total volume of 50 µl, containing 2 U of Taq DNA polymerase (Takara Biotechnology, Dalian, China). The cycling parameters for the amplification reactions were optimized for each specific fragment. The amplification products were assessed using a 1.5% TBE agarose gel stained with Goldview dye. Following purification, the products were subjected to direct sequencing in an ABI 3130 genetic analyzer.

### Statistical analyses

Statistical analyses were performed using SPSS version 19.0 for Windows (SPSS Inc., Chicago, IL, USA). To assess differences in age at diagnosis across groups, Student’s t-test was employed. For comparisons of categorical variables, either the chi-square test or Fisher’s exact test was utilized. Clinical variables that were observed in more than 5% of the patient population (*n* ≥ 20) during a univariate analysis with a significance threshold of *p* < 0.2 were subsequently included in a multivariate logistic regression analysis. The results were presented as odds ratios (ORs) with 95% confidence intervals (CIs). A two-tailed value for *p* < 0.05 was considered statistically significant.

## Results

### Cohort characteristics and classification

**Fig. 1:**
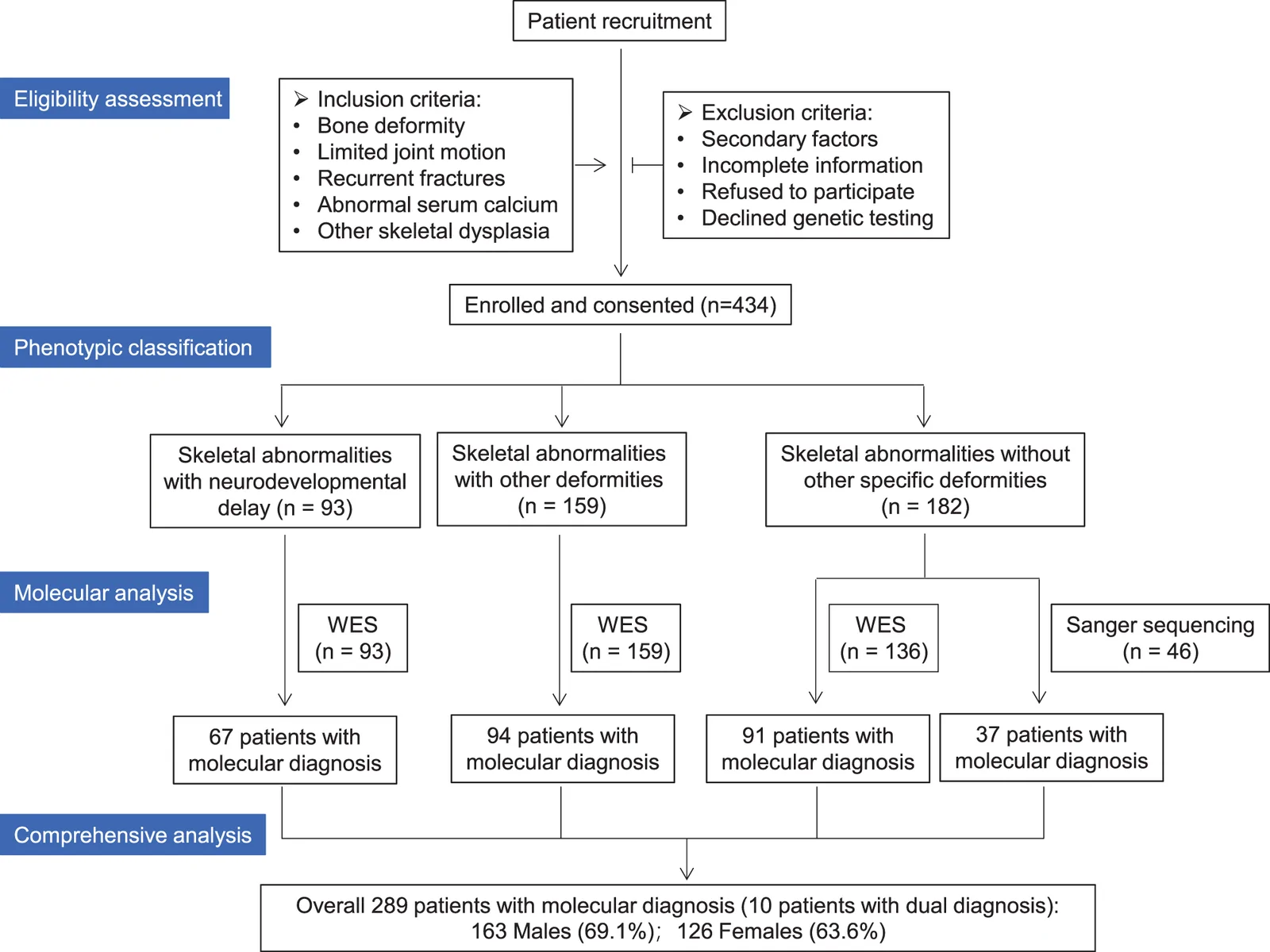
Study profile. WES, whole-exome sequencing

In our study, a cohort of 455 affected individuals from 434 families with skeletal abnormalities were investigated at the Maternal and Child Health Hospital of Guangxi Zhuang Autonomous Region (Fig. 1). Parents and siblings of probands were not considered for diagnostic yield calculation and descriptive statistical analysis. The cohort comprised 236 males (54.4%) and 198 females (45.6%). The mean age at molecular diagnosis was 5.1 years (Table 1). Notably, the mean age of diagnosis for male patients was significantly younger than that for female patients, with averages of 4.0 ± 0.39 years and 6.3 ± 0.69 years, respectively (*p* = 3.5 × 10^−3^). The patients were divided into three distinct groups based on the presence of neurodevelopmental delay or other clinical evidence for multisystem involvement: group 1 included patients with skeletal abnormalities and neurodevelopmental delay (*n* = 93); group 2 comprised patients with skeletal abnormalities accompanied by other deformities (*n* = 159); and group 3 consisted of patients with skeletal abnormalities but without other specific deformities (*n* = 182). The average age at molecular diagnosis for group 3 was significantly older than that of the other groups, recorded at 7.4 ± 0.79 years, compared to 4.4 ± 0.50 years for group 1 and 2.8 ± 0.35 years for group 2 (*p* = 1.7 × 10^−7^) (Table 1).

**Table 1:** Clinical characteristics and diagnostic results of the patients tested for whole-exome sequencing

Group	Group 1			Group 2			Group 3			Overall		
Gender	Male	Female	Total	Male	Female	Total	Male	Female	Total	Male	Female	Total
Case, No.	52	41	93	96	63	159	88	94	182	236	198	434
Age (range, median)	22d-16y, 2y6m	1m-27y, 3y	22d-27y, 3y	1d-23y, 7m	1d-24y, 8m	1d-24y, 7m	1d-40y, 2y	1d-57y, 4y	1d-57y, 3y	1d-40y, 2y	1d-57y, 3y	1d-57y, 2y
Mean, SEM (year)	4.0, 0.55	4.9, 0.89	4.4, 0.50	2.7, 0.44	2.8, 0.59	2.8, 0.35	5.4, 0.84	9.3, 1.28	7.4, 0.79	4.0, 0.39	6.3, 0.69	5.1, 0.38

Group 1, skeletal abnormalities with neurodevelopmental delay; group 2, skeletal abnormalities with other deformities; group 3, skeletal abnormalities without other specific deformities. y, year; m, month; d, day

### Molecular findings

In the context of WES analysis, a total of 67 individuals (72.0%; comprising 38 males and 29 females) from group 1 received a molecular diagnosis (Table 2, Figure S1). Among these patients, four exhibited dual molecular findings: one individual harbored a *de novo* variant in the *COL1A1* gene along with a CNV characterized by a deletion at 10q26.13-q26.3; another patient had an exon deletion in the *CREBBP* gene in conjunction with a homozygous variant in *SRD5A2*; a third individual presented with compound heterozygous variants in the *NFASC* gene and a CNV involving a deletion at 2q37.1; and the final case possessed two *NF1* variants, one inherited from the symptomatic mother and the other occurring *de novo*. Out of the 63 patients with unique molecular findings, 12.7% (eight patients) exhibited chromosomal abnormalities (including one aneuploidy and seven CNVs), 19.0% (12 patients) presented with biallelic variants (comprising two homozygous and ten compound heterozygous variants) from 12 autosomal recessive (AR) genes, and 68.3% (43 patients) were identified with a monoallelic variant (37 *de novo*, five inherited, and one unverified) from a total of 33 genes, which included 22 autosomal dominant (AD) genes, seven X-linked dominant (XD) genes, and four X-linked recessive (XR) genes (Table S1).

**Table 2:** Molecular findings of our cohort

Group, No.	Group 1			Group 2			Subgroup 3–1			Subgroup 3–2			Overall		
Gender	Male	Female	Total	Male	Female	Total	Male	Female	Total	Male	Female	Total	Male	Female	Total
**Number of cases**	52	41	93	96	63	159	67	69	136	21	25	46	236	198	434
**Molecular findings, No.**	38	29	67	60	34	94	48	43	91	17	20	37	163	126	289
**Diagnostic rate**	73.1%	70.7%	72.0%	62.5%	54.0%	59.1%	71.6%	62.3%	66.9%	81.0%	80.0%	80.4%	69.1%	63.6%	66.6%
**Dual molecular diagnosis**	4	0	4	1	1	2	1	3	4	0	0	0	6	4	10
**Single molecular diagnosis**	34	29	63	59	33	92	47	40	87	17	20	37	157	122	279
**Chromosome abnormality**	**5**	**5**	**10**	**5**	**9**	**14**	**6**	**4**	**10**	**0**	**0**	**0**	**16**	**18**	**34**
**Aneuploid**	0	1	1	1	0	1	1	0	1	0	0	0	2	1	3
**CNV**	5	4	9	4	9	13	5	4	9	0	0	0	14	17	31
**Monogenic disorder**	**37**	**24**	**61**	**56**	**26**	**82**	**43**	**42**	**85**	**17**	**20**	**37**	**153**	**112**	**265**
**AD**	18	13	31	30	13	43	27	23	50	16	20	36	91	69	160
**XD**	1	7	8	1	2	3	6	5	11	1	0	1	9	14	23
**XR**	8	0	8	11	0	11	1	0	1	0	0	0	20	0	20
**Somatic**	0	0	0	0	1	1	1	1	2	0	0	0	1	2	3
**AR**	10	4	14	14	10	24	8	13	21	0	0	0	32	27	59

Group 1, skeletal abnormalities with neurodevelopmental delay; group 2, skeletal abnormalities with other deformities; group 3–1, skeletal abnormalities without other specific deformities, the patients underwent WES analysis; group 3–2, skeletal abnormalities without other specific deformities, the patients underwent targeted Sanger sequencing; AD, autosomal dominant; XD, X-linked dominant; XR, X-linked recessive; AR, autosomal recessive

In group 2, a total of 94 patients (59.1%; consisting of 60 males and 34 females) received a molecular diagnosis by WES analysis (Table 2 and Figure S1). Among these patients, two exhibited dual molecular findings: one patient presented with a *de novo* variant in the *ZIC1* gene alongside a deletion at 16p11.2, while another harbored compound heterozygous variants in the *PCNT* gene along with two variants in the *GJB2* and *GJB6* genes. Of the 92 patients with a unique molecular diagnosis, 14.1% (13 patients) exhibited chromosomal abnormalities, which included one case of aneuploidy and 12 cases of CNVs. Additionally, 23.9% (22 patients) were found to possess biallelic variants, comprising eight homozygous and 14 compound heterozygous variants across a total of 21 AR genes. Furthermore, 62.0% (57 patients) were identified with a monoallelic variant, with 37 arising *de novo*, 14 inherited, and six unverified, spanning a total of 43 genes (32 AD genes, three XD genes, seven XR genes and one somatic gene). It is noteworthy that both AD and AR inheritance patterns of the *LMNA* gene were observed within this cohort (Table S1).

Group 3 was divided into two subgroups according to different molecular detection methodologies. Subgroup 3–1 comprised patients who underwent WES analysis (*n* = 136), while subgroup 3–2 included patients who received targeted Sanger sequencing and declined further molecular testing (*n* = 46). Within subgroup 3–1, a molecular diagnosis was established for 91 patients (66.9%), comprising 48 males and 43 females (Table 2 and Figure S1). Notably, four patients presented with dual molecular findings; three of these individuals exhibited both *de novo* variants and chromosomal abnormalities, while one patient demonstrated compound heterozygous variants in the *GALNS* gene alongside a *de novo* variant in the *CASR* gene. Among the 87 patients with unique molecular diagnoses, 8.0% (seven patients) displayed chromosomal abnormalities, 23.0% (20 patients) exhibited biallelic variants (including six homozygous and 14 compound heterozygous variants) from 14 AR genes, and 69.0% (60 patients) were identified with monoallelic variants (41 of which were *de novo*, 14 inherited, and five unverified) from a total of 26 genes (including 23 AD genes, one XD gene, one XR gene and one somatic gene) (Table S1).

Utilizing targeted Sanger sequencing, a molecular diagnosis was achieved in 81.0% of male patients and 80.0% of female patients in subgroup 3–2 (Table 2 and Figure S1). A total of 34 variants in the *FGFR3* gene, one variant in the *FGFR2* gene, one variant in the *COL1A1* gene, and one nonsense variant in the *PHEX* gene were identified. The most abundant pathogenic variant identified was p.G380R in the *FGFR3* gene, accounting for 82.4% of the detected alleles (Table S2).

### Inheritance patterns and pathogenicity classification of genetic variants

**Fig. 2:**
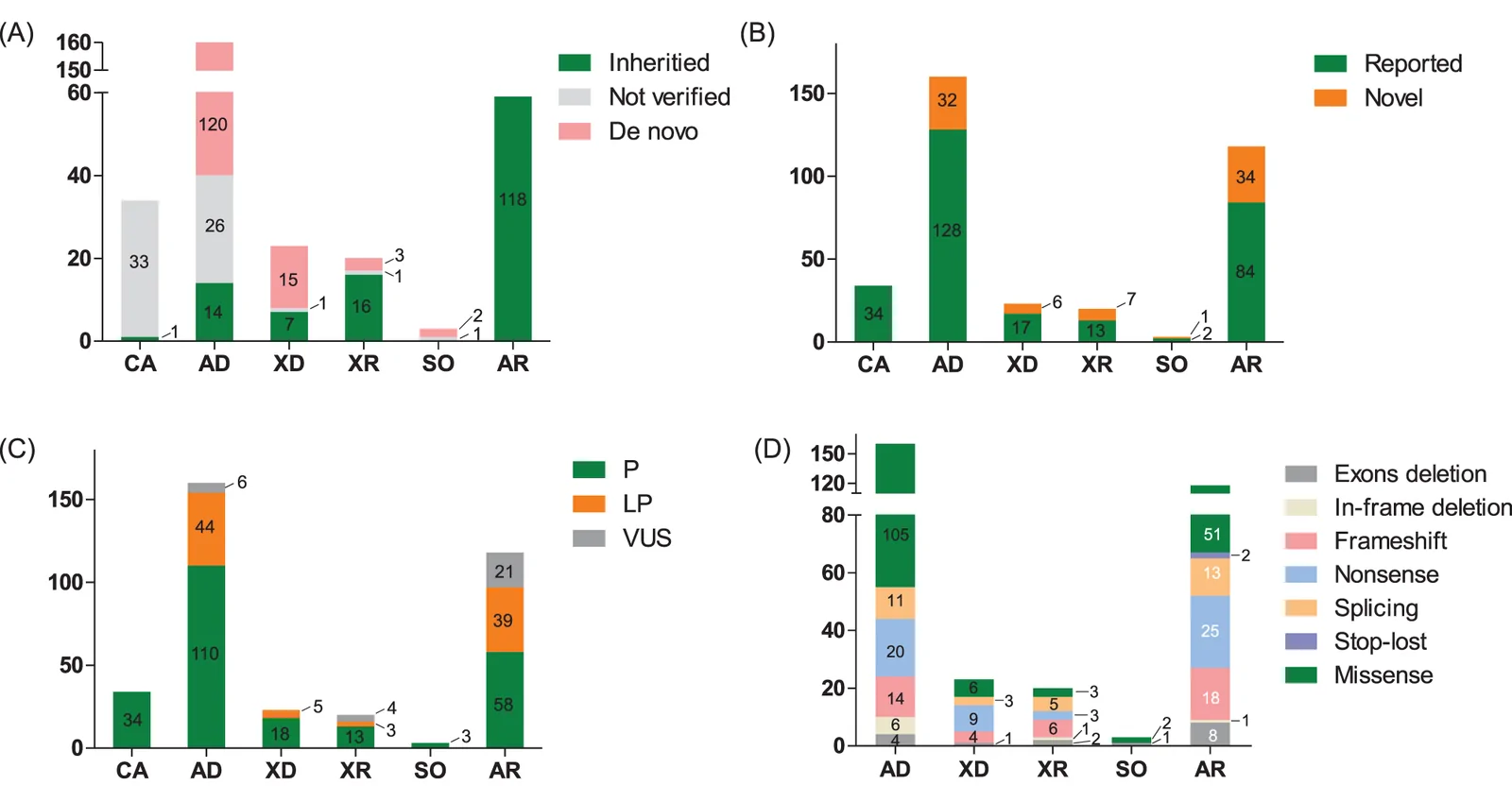
Diagnostic variants were identified in our cohort. (**A**) Inheritance patterns among the diagnostic variants. (**B**) Novelty of identified variants. (**C**) the classification of pathogenicity for variations according to the ACMG/AMP variant classification guidelines. (**D**) Variant types among diagnostic variants of monogenic diseases. CA, chromosomal abnormalities; AD, autosomal dominant; XD, X-linked dominant; XR, X-linked recessive; SO, somatic; AR, autosomal recessive. P, pathogenic; LP, likely pathogenic; VUS, variant of uncertain significance. The number of variants was contingent upon the frequency of allelic alterations observed in the patients

In this study, all identified chromosomal abnormalities, which included three instances of aneuploidy and 31 CNVs, were documented as pathogenic (Fig. 2A–B). Notably, one CNV was traced back to the symptomatic father of the patient (family SD-3–123). Among the 160 genetic variants associated with AD inheritance, comprising 128 previously reported and 32 novel variants, a substantial proportion, amounting to 120 variants (75.0%), were classified as *de novo*. Additionally, 14 variants (8.8%) were identified as having either paternal or maternal origins; of these, 12 exhibited clinical features, while two lacked discernible characteristics. According to the ACMG/AMP guidelines for variant interpretation, 110 variants were classified as pathogenic, 44 as likely pathogenic, and six as VUS, all of which were missense variants (Fig. 2A–C). Furthermore, the 23 identified variants associated with XD inheritance, which included 17 previously reported and six novel variants, comprised 18 pathogenic variants and five variants that may be classified as likely pathogenic. Among these, 15 variants (65.2%) were *de novo*, while six (26.1%) were of paternal or maternal origin; four exhibited clinical features, and two lacked obvious characteristics. Additionally, a missense variant in the *EBP* gene was inherited from an unaffected chimeric father (Fig. 2A–C).

The majority of X-linked recessive genetic variations, which included 13 previously reported and seven novel variants, are predominantly inherited through the maternal lineage, accounting for 16 out of 20 individuals. This group includes 13 pathogenic variants, three likely pathogenic variants, and four VUS (Fig. 2A–C). Additionally, two instances of *de novo* pathogenic variants in the *PIK3CA* gene, indicative of somatic inheritance, have been reported, along with a novel variant resulting from an exon deletion derived from an unverified source. Furthermore, among the 118 identified autosomal recessive genetic variants, which encompass 34 novel variants, 97 were classified as pathogenic or likely pathogenic, while 21 were considered as VUS (20 missense variants and one splicing variant) (Fig. 2A–C).

### Types of mutations in monogenic disorders

Missense mutations represented the predominant category of genetic variations associated with AD inheritance, comprising 105 out of 160 cases (65.6%), and AR inheritance, accounting for 51 out of 118 cases (43.2%). This was followed by truncating mutations, which included splicing variants, nonsense mutations, and frameshift mutations, representing 28.1% and 47.5% of the respective inheritance patterns. In the context of X-linked genetic diseases, truncated variants constituted 69.6% (16 out of 23) of XD variants and 70.0% (14 out of 20) of XR variants (Fig. 2D).

### Diagnostic efficiency analysis

In this study, the molecular diagnostic rate associated with Sanger sequencing demonstrated a slight superiority over that of WES analysis, with rates of 80.4% and 64.9% (*p* = 0.035), respectively (Table S3). Notably, the diagnostic rates for WES did not differ significantly between male and female patients, reporting rates of 67.9% and 61.3%, respectively (*p* = 0.173), as shown in Table 2 and Table S3. Additionally, the molecular diagnostic rate for group 2 was significantly lower than that of group 1, with rates of 59.1% and 72.0% (*p* = 0.039), respectively (Table S3 and Figure S1). However, the rate for group 2 was only slightly lower than that of subgroup 3–1, which had a rate of 66.9%, and this difference was not statistically significant (*p* = 0.168) (Table S3). Furthermore, the diagnostic rates observed for Proband-WES (228/346, 65.9%) and Trio-WES (24/42, 57.1%) did not exhibit a statistically significant difference (*p* = 0.262) (Table S1).

**Fig. 3:**
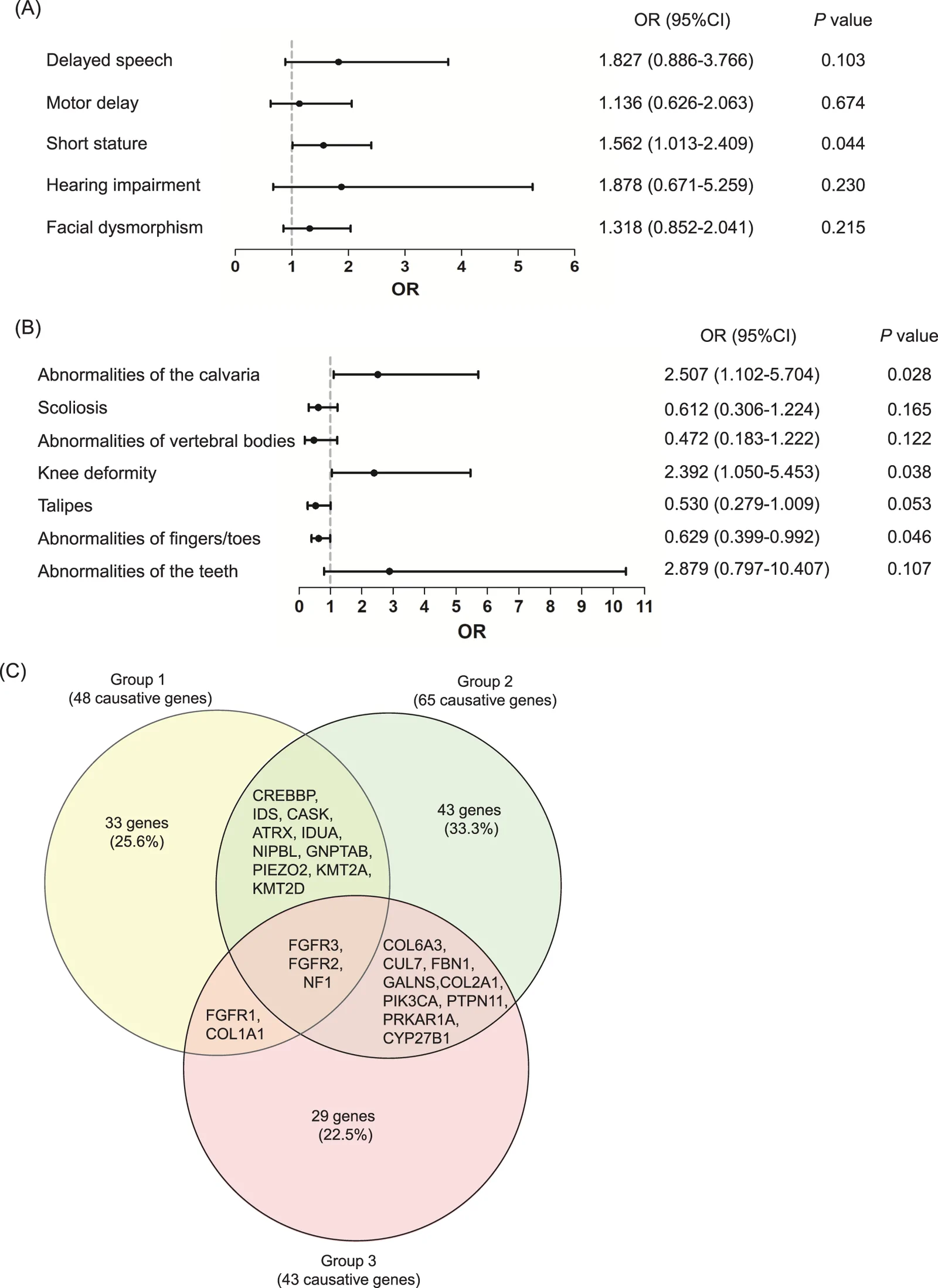
Diagnostic results of different clinical features. (**A**) various clinical phenotypes of the extra-skeletal system were analyzed using a logistic regression model. OR, odds ratio; 95% CI, 95% confidence interval. (**B**) various clinical phenotypes of the skeletal system were assessed using a logistic regression model. (**C**) distribution of diagnostic genes across different clinical features. Yellow circle represents skeletal abnormalities with neurodevelopmental delay; green circle indicates skeletal abnormalities with other deformities; red circle signifies isolated skeletal abnormalities without other specific deformities

In the cohort of patients who underwent WES analysis, the predominant clinical manifestations observed were facial dysmorphism and short stature, with growth retardation and motor delay also being prevalent (Table 3). We conducted an analysis of the molecular diagnostic rates among affected individuals (*n* ≥ 20) exhibiting various clinical phenotypes who underwent WES. Our findings indicated that short stature (*p* = 0.044, odds ratio [OR] [95% confidence interval {CI}] = 1.562 [1.013–2.409]) serves as a significant predictive factor for obtaining a positive molecular finding (Fig. 3A).

**Table 3:** Clinical characteristics and diagnostic results of the patients tested for whole-exome sequencing

Clinical characteristics	Affected individuals, n (%)	Diagnosed individuals, n (%)	p value
Abnormality of the calvaria	42 (10.8%)	34 (81.0%)	0.021
Laryngeal cartilage malformation	5 (1.3%)	3 (60.0%)	1.000
Abnormality of the scapula	6 (1.5%)	3 (50.0%)	0.427
Abnormality of the thorax	51 (13.1%)	33 (64.7%)	0.936
Abnormality of the ribs	20 (5.2%)	11 (55.0%)	0.325
Scoliosis	44 (11.3%)	24 (54.5%)	0.125
Abnormality of the vertebral bodies	21 (5.4%)	10 (47.6%)	0.087
Short long bones	88 (22.7%)	60 (68.2%)	0.505
Abnormality of long bones	20 (5.2%)	15 (75.0%)	0.345
Abnormality of the hip joint	15 (3.9%)	11 (73.3%)	0.500
Abnormality of the elbow	12 (3.1%)	8 (66.7%)	0.914
Knee deformity	42 (10.8%)	34 (81.0%)	0.021
Talipes	47 (12.1%)	25 (53.2%)	0.072
Other joint abnormalities	49 (12.6%)	35 (71.4%)	0.328
Abnormality of the hand	44 (11.3%)	31 (70.5%)	0.438
Abnormality of the foot	27 (7.0%)	16 (59.3%)	0.501
Abnormalities of fingers/toes	142 (36.6%)	85 (59.9%)	0.060
Abnormalities of the teeth	20 (5.2%)	17 (85.0%)	0.054
Bone fracture	15 (3.9%)	10 (66.7%)	0.904
Osteoporosis	21 (5.4%)	12 (57.1%)	0.425
Osteoma	3 (0.8%)	3 (100.0%)	0.555
Intellectual disability	67 (17.3%)	48 (71.6%)	0.207
Delayed speech and language development	74 (19.1%)	57 (77.0%)	0.015
Motor delay	114 (29.4%)	83 (72.8%)	0.036
Short stature	193 (49.7%)	136 (70.5%)	0.023
Growth retardation	144 (37.1%)	91 (63.2%)	0.578
Abnormality of body weight	29 (7.5%)	16 (55.2%)	0.251
Feeding difficulties	16 (4.1%)	12 (75.0%)	0.401
Abnormality of the respiratory system	15 (3.9%)	9 (60.0%)	0.666
Dystonia	70 (18.0%)	46 (65.7%)	0.882
Muscular weakness	17 (4.4%)	11 (64.7%)	0.965
Seizures	24 (6.2%)	17 (70.8%)	0.533
Ataxia	12 (3.1%)	7 (58.3%)	0.612
Hearing impairment	24 (6.2%)	19 (79.2%)	0.132
Premature birth	25 (6.4%)	11 (44.0%)	0.023
Facial dysmorphism	194 (50.0%)	136 (70.1%)	0.033
Abnormality of the nail	8 (2.1%)	4 (50.0%)	0.459
Abnormality of brain morphology	50 (12.9%)	33 (66.0%)	0.867
EEG abnormality	4 (1.0%)	3 (75.0%)	1.000
Behavioral abnormality	12 (3.1%)	9 (75.0%)	0.554
Abnormal dermatoglyphics	21 (5.4%)	14 (66.7%)	0.865
Abnormality of skin	46 (11.9%)	30 (65.2%)	0.999
Edema	3 (0.8%)	3 (100.0%)	0.555
Abnormality of the endocrine system/metabolism	54 (13.9%)	35 (64.8%)	0.948
Abnormality of the kidney	10 (2.6%)	7 (70.0%)	0.747
Abnormality of the heart	54 (13.9%)	37 (68.5%)	0.582
Abnormality of pulmonary situs	3 (0.8%)	0 (0.0%)	0.042
Abnormality of the genital system	34 (8.8%)	20 (58.8%)	0.433
Abnormality of the urinary system	14 (3.6%)	6 (42.9%)	0.089
Abnormality of the ocular region	19 (4.9%)	11 (57.9%)	0.493
Abnormality of the liver/spleen	11 (2.8%)	9 (81.8%)	0.341
Abnormality of the digestive system	12 (3.1%)	5 (41.7%)	0.120
Hemopathy	19 (4.9%)	13 (68.4%)	0.763
Hernia	17 (4.4%)	13 (76.5%)	0.319
Abnormality of the amniotic fluid	11 (2.8%)	6 (54.5%)	0.451

EEG, electroencephalogram

Among the phenotypic manifestations associated with SD, the occurrence of digit abnormalities and the presence of shortened long bones are particularly significant (Table 2). In these characteristics, abnormalities of the calvaria (*p* = 0.028, OR [95% CI] = 2.507 [1.102–5.704]) and knee deformities (*p* = 0.038, OR [95% CI] = 2.392 [1.050–5.453]) emerged as significant predictive factors for a positive molecular diagnosis (Fig. 3B).

### Genetic spectrum

In our study cohort, we identified a total of 34 chromosomal abnormalities and 265 Mendelian genetic disorders associated with skeletal anomalies (Table 1 and Table S1–S2). Across the three categories analyzed, 129 causative genes were identified, with three genes (*FGFR3*, *FGFR2* and *NF1*) present in all three groups (Fig. 3C).

**Fig. 4:**
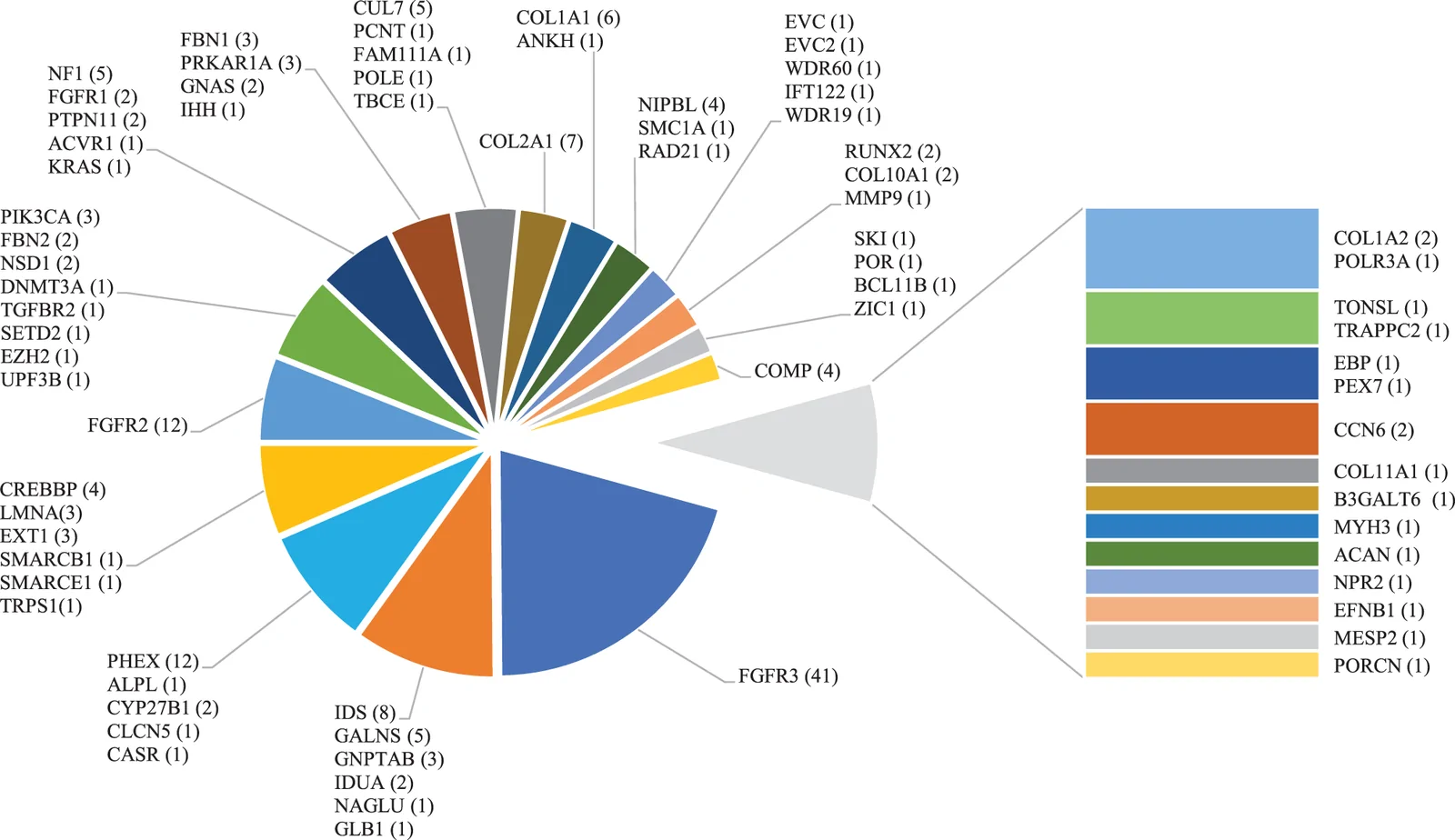
Spectrograms of causative genes listed in the Nosology of genetic skeletal disorders: 2023 revision. The colors represent different disease groups. The number of patients for each gene is indicated in parentheses

According to the 2023 revision of the Nosology of genetic skeletal disorders, we identified 75 causal genes distributed across 32 groups from a total of 199 positive molecular diagnoses (illustrated in Fig. 4 and detailed in Table S4). The *FGFR3* gene was the most frequently identified causal gene, accounting for 41 alleles (20.6%), followed by *FGFR2* (12 alleles, 6.0%), *PHEX* (12 alleles, 6.0%), *IDS* (8 alleles, 4.0%), *COL2A1* (7 alleles, 3.5%), *COL1A1* (6 alleles, 3.0%), *CUL7* (5 alleles, 2.5%), *GALNS* (5 alleles, 2.5%), and *NF1* (5 alleles, 2.5%). Collectively, these genes accounted for over half of all diagnoses, totaling 50.8%.

**Fig. 5:**
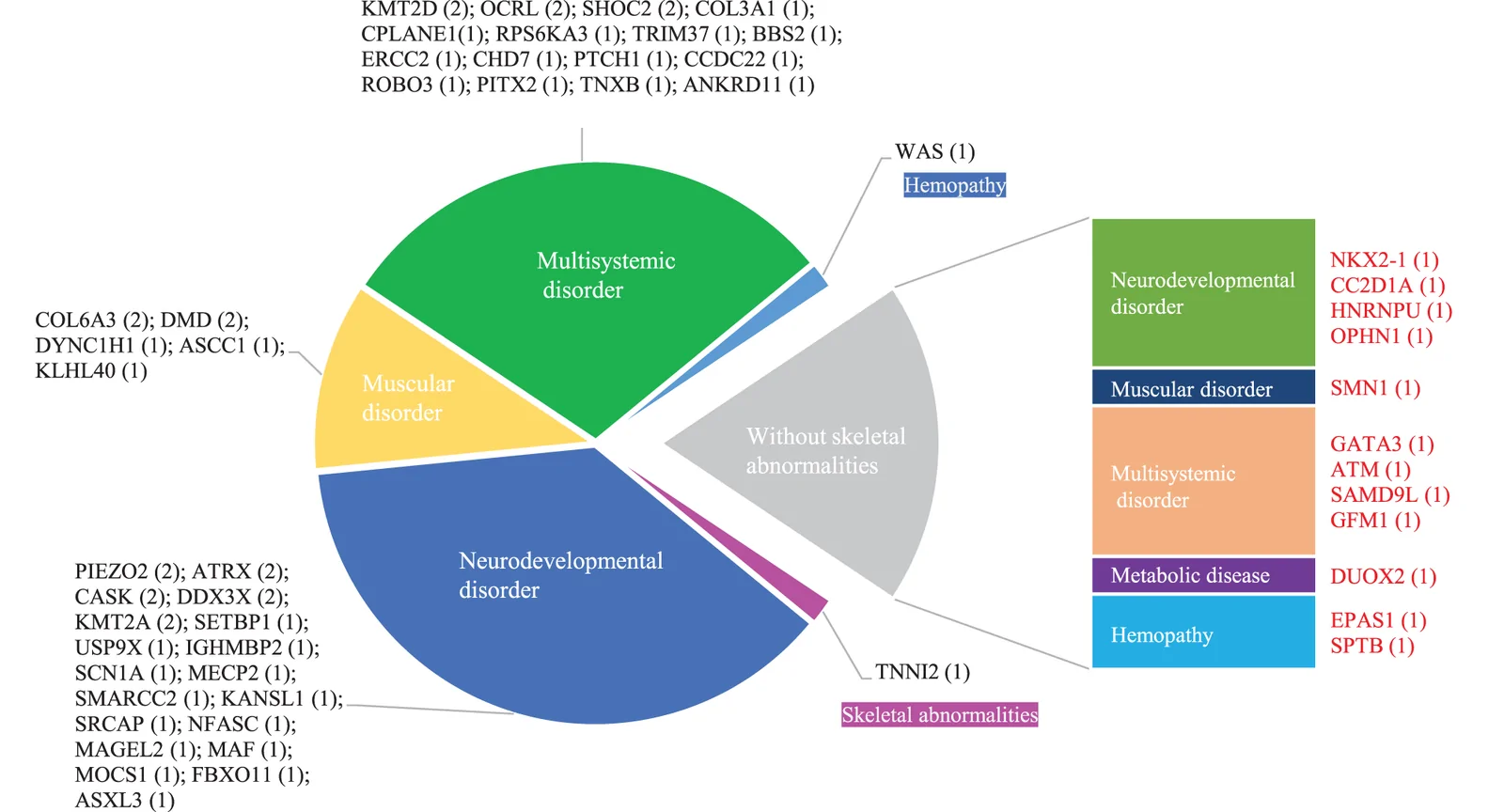
Spectrograms of causative genes not listed in the Nosology of genetic skeletal disorders: 2023 revision. The colors represent different disease categories. The number of patients for each gene is indicated in parentheses. Genes without skeletal abnormalities are highlighted in red font

The remaining 66 positive diagnoses were responsible for an additional 56 causal genes (Fig. 5 and Table S5). Notably, two mutations were identified in 10 different genes, each representing 3.1% (2 cases) for the respective gene. The *SRD5A2* variants are associated with pseudovaginal perineoscrotal hypospadias, whereas the *GJB2*/*GJB6* variants are linked to deafness. This information contributes to a more thorough understanding of the patient’s phenotypic presentation. Of the 54 remaining causal genes, 42 have been documented in the OMIM database as being linked to skeletal abnormalities. This group includes one gene associated with skeletal abnormalities (*TNNI2* gene), 19 related to neurodevelopmental disorders, 16 associated with multisystemic disorders, five connected to muscular disorders, and one associated with hemopathy. The remaining 12 genes, which encompass four associated with neurodevelopmental disorders, four linked to multisystemic disorders, one related to a muscular disorder, one associated with metabolic disease, and two connected to hemopathies, have not been reported as related to SD.

### Asymptomatic individuals with mutations

An analysis was performed on all identified pathogenic/likely pathogenic variants related to the latest version of genetic skeletal disorders within our in-house database. During the study period, a total of 100 variants across 49 causative genes were identified in 99 patients who exhibited no associated clinical manifestations. Notably, one patient harbored mutations in both the *NF1* and *MYCN* genes (Table S6).

## Discussion

SD encompass a wide range of diseases characterized by intricate clinical phenotypes and pathogenic mechanisms. Studies involving large cohorts are crucial for gaining insights into disease epidemiology, phenotype profiles, and mutation characteristics. This study represents one of the most extensive investigations conducted on an unselected cohort of patients involving SD. A total of 34 chromosomal abnormalities and 265 Mendelian genetic disorders were identified. The overall diagnostic yield from WES analysis in our cohort was 64.9%, indicating the efficacy of WES in achieving molecular diagnoses for individuals with SD. Furthermore, the detection efficiency of Sanger sequencing has achieved an impressive rate of 80.4%. Our research focused on investigating the clinical and genetic profiles of patients with SD in Guangxi, thereby providing valuable clinical experience for the effective implementation of genetic testing in the context of SD within our region.

Previous postnatal WES assessments have demonstrated diagnostic yields ranging from 25% to 30% in individuals with suspected genetic disorders. The efficacy of WES in identifying pathogenic variants differs across various disease categories [9, 10]. The diagnostic yield in this study exceeds those reported in several prior investigations, including Najjar et al., 2025 (50%); Scocchia et al., 2021 (42%); Lv et al., 2021 (49%); Maddirevula et al., 2018 (44%); Bae et al., 2016 (52%); Retterer et al., 2016 (39%) and Zhang et al., 2015 (54%) [1, 2, 11–15]. Conversely, other investigations, such as those conducted by Jacob et al. (2025) and Silveira et al. (2021), have reported higher diagnostic rates of 71 and 77%, respectively [16, 17]. Several factors can influence diagnostic efficacy, including patient selection criteria, case selection, diagnostic methodologies employed, sample sizes, whether analyses are conducted on probands alone or in trio configurations, and other variables (Table S7). In this research, WES and targeted Sanger sequencing were utilized to identify causative variants. Notably, no statistically significant difference in diagnostic rates was observed between proband-only WES and trio-based WES within our cohort. This outcome may be attributed to the limited number of trios incorporated. Additionally, the detection rate was slightly higher among patients exhibiting isolated skeletal abnormalities or skeletal abnormalities alongside neurological abnormalities compared to those presenting with skeletal abnormalities accompanied by multiple deformities. This difference may be attributed to the elevated positive diagnostic yield of WES in individuals with neurological disorders [9].

Utilizing the coverage depth of capture sequencing data, WES analysis demonstrated the capability to detect chromosomal abnormalities, including CNVs, thereby significantly improving diagnostic yield [18]. In this investigation, chromosomal abnormalities accounted for 11.4% (34 out of 299) of all diagnostic findings. Notably, several CNVs were predominantly linked to skeletal deformities, including the deletion at Xp22.33, impacting the *SHOX* gene (families SD-2–136 and SD-3–048), and the deletion at 17p13.3 involving the *BHLHA9* gene (family SD-3–123). These findings underscore the ongoing significance of analyzing chromosomal abnormality analysis in the diagnosis of skeletal disorders. Similarly, WES analysis enabled the detection of whole gene deletions as well as deletions encompassing one or more exons. This study identified a total of 14 exon deletions that were causally linked to the observed manifestations (Fig. 2D). Additionally, with adequate sequencing depth, it is feasible to detect mosaic mutations that may contribute to disease pathogenesis. In this cohort, three mosaic pathogenic variants were identified, specifically two in the *PIK3CA* gene and one in the *PHEX* gene. Furthermore, the identification of patients with dual diagnoses enhances symptom interpretation and facilitates precise therapeutic interventions. This study identified 10 patients with dual diagnoses, including six cases presenting simultaneous chromosomal abnormalities and Mendelian genetic disorders.

The utilization of next-generation sequencing technology has the potential to yield a variety of VUS. The VUS associated with patients’ clinical characteristics pose considerable challenges for clinical interpretation and genetic counseling. In this study, 31 VUS were identified as contributing to a positive molecular diagnosis, with a notable majority being missense mutations (27 out of 31). Additionally, in-frame deletions and non-canonical splice site variants have also been implicated. Beyond the variant type, their pathogenicity is influenced by several factors, including the absence of parental testing, inheritance from an unaffected parent exhibiting incomplete penetrance, or adherence to an X-linked recessive inheritance pattern. To comprehensively elucidate the pathogenic mechanisms associated with these variants, further functional experiments, additional family studies, and long-term follow-up are warranted. For example, in family SD-3–017, the aberrant splicing of a non-canonical splice site variant in the *ROBO3* gene was verified through RNA analysis, leading to the reclassification of the variant from uncertain significance to likely pathogenic [19]. The pathogenicity of some VUS is presently undergoing validation via functional experiments. Furthermore, it is essential to remain informed about the latest advancements in research and to promote data sharing. Prior to the reclassification of VUS, it is imperative for genetic counselors to provide a comprehensive explanation of VUS to patients. This strategy aims to mitigate potential psychological distress and to avoid unwarranted medical interventions.

It is essential for genetic counselors and clinical practitioners to clearly articulate the scope and limitations associated with WES testing [20]. In cases where patients receive negative outcomes from molecular testing, the underlying genetic factors may remain ambiguous, or certain genetic aberrations may not be detectable through WES. Such aberrations may include structural variants, deep intronic variants, and alterations in regions characterized by repetitive sequences (e.g., the collagen genes) or high GC content (such as the translation initiation region of some genes, including the *EVC* gene) [1, 15, 21]. In these instances, supplementary molecular assays or targeted biochemical indicators may be employed to establish a definitive diagnosis. Additionally, it is crucial for geneticists to pursue further investigations and routinely reanalyze uncertain or negative results, as this practice is vital for enhancing diagnostic efficacy.

WES analysis is generally recognized for its superior diagnostic efficiency compared to Sanger sequencing, primarily attributed to its extensive gene coverage [11]. Interestingly, in the present study, the molecular diagnostic rate for targeted gene sequencing was marginally higher than that for WES analysis. Jacob et al. reported a significant diagnostic rate of 74% (37/50) for targeted gene sequencing in patients with SD [16]. However, it is important to note that both investigations were conducted at a single center with a limited sample size, necessitating further validation through larger, multicenter studies. Targeted Sanger sequencing continues to be a valuable methodology for identifying causal variants associated with recurrent diseases that exhibit distinct clinical symptoms and involve specific candidate genes, such as *FGFR3*-related disorders. Moreover, utilizing Sanger sequencing to assess the predominant pathogenic genes associated with SD represents a cost-effective strategy. However, the effectiveness of this strategy is contingent upon the clinician’s accurate clinical data regarding affected individuals and a comprehensive understanding of skeletal disorders to appropriately select candidate genes.

In the context of dominant genetic disorders attributable to *de novo* variants, the recurrence risk within a family is generally considered to be low. However, it is essential to consider the potential impact of parental mosaicism. For instance, *EBP* mutations are associated with X-linked dominant chondrodysplasia punctata-2 (MIM#302960), with affected males predominantly resulting from postzygotic mosaicism involving an *EBP* variant [22]. In the case of family SD-2–056, the father of the proband exhibited no clinical symptoms; nevertheless, approximately 20% of the sequencing reads obtained from peripheral blood were positive for the mutation. This finding implies that varying chimeric states within the organism may result in diverse clinical presentations. Additionally, in family SD-3–065, a pathogenic variant in the *COMP* gene was detected in all three siblings, yet it was absent in the leukocyte DNA of either parent, indicating the possibility of parental germline mosaicism.

In our cohort, in addition to SD, the most prevalent clinical manifestations among patients included facial dysmorphism and short stature, followed by growth retardation and motor delay. The findings indicated a significant correlation between these clinical features and abnormal skeletal development. It should be noted that this outcome may be influenced by selection bias, as our hospital functions as a regional center for the treatment of short stature and developmental disorders. Furthermore, individuals with short stature, calvarial abnormalities or knee deformities demonstrated a greater likelihood of revealing a molecular etiology through WES. These findings may provide valuable clinical indicators for the implementation of genetic testing in patients with SD.

The delayed implementation of molecular diagnosis raises significant concerns. A similar pattern was noticed in the research conducted by Li et al. at another medical institution in China [23]. The discrepancy in the age of molecular diagnosis between groups indicates that patients with isolated skeletal abnormalities have not yet received adequate attention. There is an urgent necessity for timely diagnosis and intervention for individuals presenting with cognitive impairments or multiple malformations [24]. The observed difference in the mean age of diagnosis between male and female patients may be attributed to delays in genetic counseling and molecular diagnosis for certain female patients until reproductive options were contemplated. In our cohort, among patients aged 18 and older, there were 24 females and seven males (11.6% vs. 3.0%, *p* = 4.03 × 10^−4^). Additionally, the economic burden associated with molecular testing has significantly contributed to delays in the diagnosis and treatment of genetic skeletal disorders, particularly in middle- and low-income regions. Achieving timely diagnoses and ensuring access to appropriate treatments for these conditions necessitates heightened public awareness, increased attention, and greater financial support. Early intervention is known to be more beneficial for SD patients. For instance, administration of recombinant human growth hormone (rhGH) has been demonstrated to effectively improve the height of children with achondroplasia within a five-year period [25]. Additionally, treatment with phosphate salts and active vitamin D has been shown to significantly improve the prognosis for children with hypophosphatemic rickets [26]. A delayed diagnosis may result in patients missing the optimal window for the initiation of effective treatment. Furthermore, for the families of affected individuals, a delayed diagnosis may have implications for their reproductive planning and increase the risk of reproducing offspring with inherited disorders.

The nosology is intended assist pediatricians, geneticists, and radiologists in achieving precise diagnoses for their patients, while also facilitating advancements in skeletal biology research. In this study, approximately 75% of the monogenic disorders (199/265) identified are documented in the 2023 edition of the Nosology of genetic skeletal disorders (Table S4). The nosology and classification of genetic skeletal disorders have significantly improved with each edition, particularly over the last decade, largely due to the utilization of high-throughput sequencing in medical genetics research and diagnostics [7]. The categorization and nomenclature of diseases are continuously refined as novel causative genes are consistently identified. In our research, the *PORCN*, *ZIC1*, and *BCL11B* genes were absent from the 2019 edition of the Nosology, but were included in the 2023 edition [7, 27]. These advancements stem from extensive clinical studies and molecular diagnoses of skeletal disorders. Our research has identified 56 genes that have not yet been classified as causative for inherited bone diseases (Fig. 5, Table S5). With the exception of the *SRD5A2* and *GJB2*/*GJB6* genes, which contribute to a more comprehensive understanding of the complex phenotypes observed in patients (dual diagnosis), the remaining 54 genes may be implicated in SD. Further research and data collection are necessary to validate any potential associations.

It is important to acknowledge that this research, as a retrospective single-center study, may be subject to inherent biases associated with such a study design. Consequently, the results may not accurately reflect the genetic architecture of skeletal disorders across different populations. Additionally, the study’s phenotype-driven inclusion criteria may have resulted in the omission of asymptomatic individuals with inherited bone diseases. The *NF1* and *PTPN11* genes are categorized within the disorganized development of skeletal components group (group 30) [7]. In our local database, only four patients with *NF1* pathogenic variants exhibited pectus carinatum, abnormal joint morphology, or lower limb asymmetry, while 19 patients had not yet presented with SD (Table S6). Additionally, two patients with *PTPN11* mutations presented with either pectus carinatum or spondyloepimetaphyseal dysplasia, whereas the remaining six exhibited no signs of SD. Moreover, an *FGFR3* variant was detected in a newborn, and *COL1A1* variants were identified in both a child and a newborn; however, none of these individuals exhibited skeletal abnormalities, and these variants were deemed “unexpected findings”. These results further illustrate the significant clinical heterogeneity of genetic skeletal disorders and the intricacies involved in their clinical diagnosis. In addition to standardized clinical evaluations, biochemical testing, and radiographic assessments, these “unexpected findings” underscore the critical role of molecular testing for individuals with skeletal dysplasia, particularly in fetuses and newborns. Definitive molecular diagnosis has the potential to modify medical management and improve treatment outcomes.

## Conclusions

This study has made substantial contributions to understanding the clinical and genetic characteristics of SD by conducting a comprehensive analysis of 434 affected families in Guangxi. It has also elucidated the mutation spectrum of the causative genes associated with this condition in the region. Additionally, the research has validated the effectiveness of WES as a molecular diagnostic tool for individuals with suspected SD. Large-scale clinical and molecular investigations facilitate the establishment of gene-disease relationships, while also contribute to the assessment of disease severity and the prediction of patient outcomes.

## Electronic supplementary material

Below is the link to the electronic supplementary material.


Supplementary material 1



Supplementary material 2



Supplementary material 3



Supplementary material 4



Supplementary material 5



Supplementary material 6



Supplementary material 7



Supplementary material 8


## Data Availability

The data that support the findings of this study are available upon request to the corresponding author.

## Abbreviations

SD: Skeletal dysplasia
WES: Whole-exome sequencing
CNV: Copy number variants
MLPA: Multiplex ligation-dependent probe amplification
ACMG/AMP: American College of Medical Genetics and Genomics and the Association for Molecular Pathology
AD: Autosomal dominant
XD: X-Linked dominant
XR: X-Linked recessive
AR: Autosomal recessive
VUS: Variant of uncertain significance
