# Supplementary material for: Clinical and genetic profiles of postnatal patients with skeletal dysplasia in Guangxi during 8 years: a single-center experience
Source: Orphanet J Rare Dis. 2026 Aug 31;21:305. doi: 10.1186/s13023-026-04414-2 (PMC13528159; doi:10.1186/s13023-026-04414-2)
Supplement: Supplementary file 1 [file 13023_2026_4414_MOESM1_ESM.pdf]

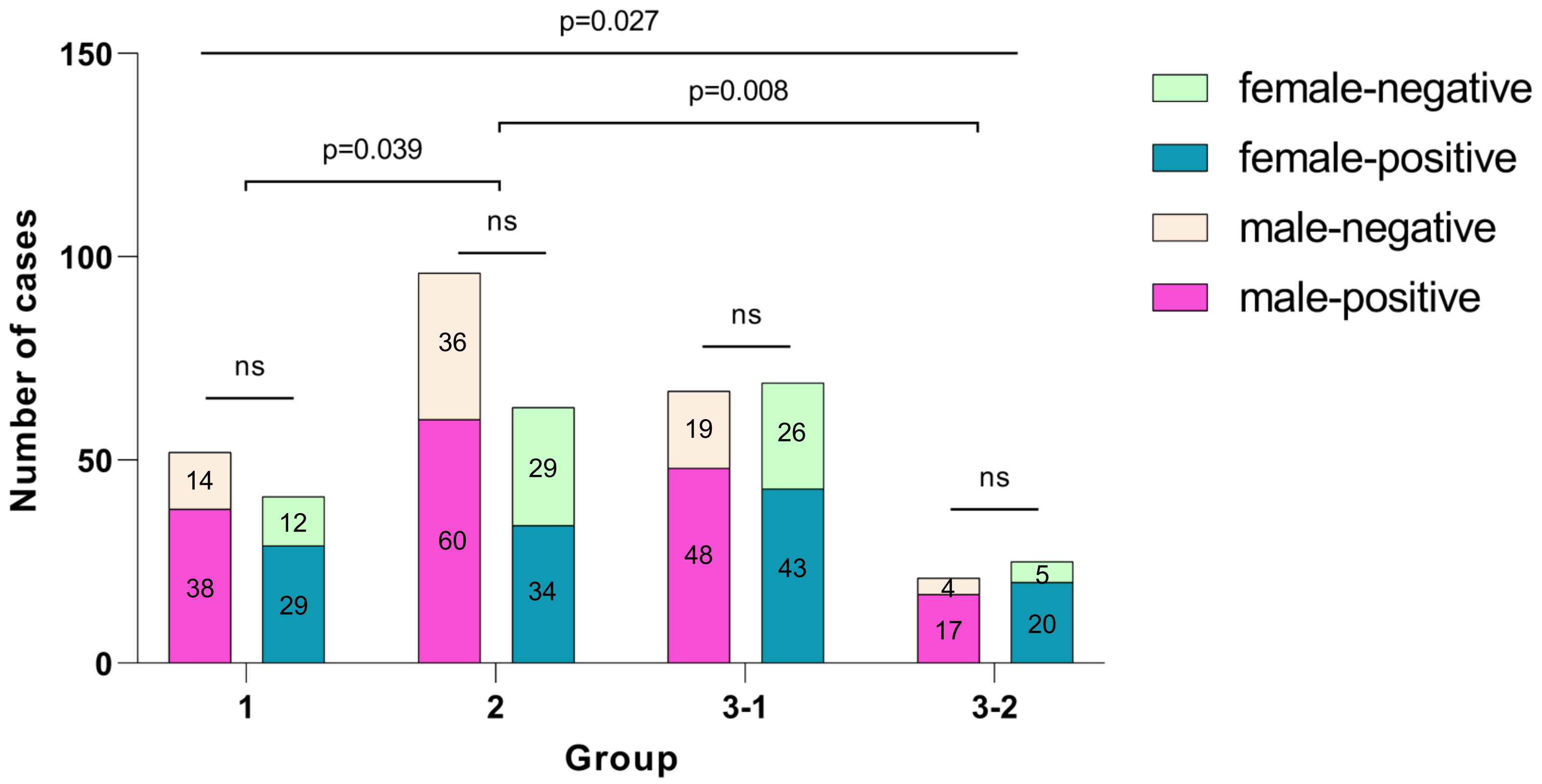
p=0.027
p=0.008
p=0.039
ns
ns
ns
ns
150
100
50
0
Number of cases
14
38
12
29
36
60
29
34
19
48
26
43
4
17
5
20
1
2
3-1
3-2
Group
female-negative
female-positive
male-negative
male-positive
