# Supplementary material for: Clinical and genetic profiles of postnatal patients with skeletal dysplasia in Guangxi during 8 years: a single-center experience
Source: Orphanet J Rare Dis. 2026 Aug 31;21:305. doi: 10.1186/s13023-026-04414-2 (PMC13528159; doi:10.1186/s13023-026-04414-2)
Supplement: Supplementary file 2 [file 13023_2026_4414_MOESM4_ESM.docx]

Table S6 Comparison of detection rates.

|  | **Pearson Chi-Square** | ***P* value** |
| --- | --- | --- |
| male *vs.* female |  |  |
| Group 1 | 0.063 | 0.802 |
| Group 2 | 1.146 | 0.284 |
| Group 3-1 | 1.334 | 0.248 |
| Group 3-2 | 0.007 | 1.000 |
| WES | 1.854 | 0.173 |
| Total | 1.428 | 0.232 |
| Group |  |  |
| group 1 *vs.* group 2 | 4.248 | 0.039 |
| group 1 *vs.* group 3-1 | 0.68 | 0.410 |
| group 1 *vs.* group 3-2 | 1.15 | 0.283 |
| group 2 *vs.* group 3-1 | 1.903 | 0.168 |
| group 2 *vs.* group 3-2 | 7.027 | 0.008 |
| group 3-1 *vs.* group 3-2 | 3.012 | 0.083 |
| WES vs SS | 4.433 | 0.035 |
| Total | 9.201 | 0.027 |

WES, whole-exome sequencing; SS, Sanger sequencing.
