# Supplementary material for: Clinical and genetic profiles of postnatal patients with skeletal dysplasia in Guangxi during 8 years: a single-center experience
Source: Orphanet J Rare Dis. 2026 Aug 31;21:305. doi: 10.1186/s13023-026-04414-2 (PMC13528159; doi:10.1186/s13023-026-04414-2)
Supplement: Supplementary file 3 [file 13023_2026_4414_MOESM5_ESM.docx]

Table S3. The number of patients with causative genes listed in the Nosology of genetic skeletal disorders: 2023 revision.

| **Causative gene** | **Classification of skeletal disorders** | **Number of cases** | **Proportion** |
| --- | --- | --- | --- |
| *FGFR3* | 1, 31, 34, 40 | 41 | 20.60% |
| *COL2A1* | 2 | 7 | 3.52% |
| *COL11A1* | 3 | 1 | 0.50% |
| *B3GALT6* | 5 | 1 | 0.50% |
| *MYH3* | 6 | 1 | 0.50% |
| *ACAN* | 7 | 1 | 0.50% |
| *COMP* | 9 | 4 | 2.01% |
| *EVC2* | 10, 35 | 1 | 0.50% |
| *EVC* | 10, 35 | 1 | 0.50% |
| *WDR60* | 10 | 1 | 0.50% |
| *IFT122* | 10 | 1 | 0.50% |
| *WDR19* | 10 | 1 | 0.50% |
| *RUNX2* | 11, 33, 34 | 2 | 1.01% |
| *COL10A1* | 11 | 2 | 1.01% |
| *MMP9* | 11 | 1 | 0.50% |
| *TONSL* | 13 | 1 | 0.50% |
| *TRAPPC2* | 13 | 1 | 0.50% |
| *NPR2* | 16, 31 | 1 | 0.50% |
| *FBN1* | 17, 31 | 3 | 1.51% |
| *PRKAR1A* | 17 | 3 | 1.51% |
| *GNAS* | 17, 30 | 2 | 1.01% |
| *IHH* | 17, 18, 40 | 1 | 0.50% |
| *CREBBP* | 19 | 4 | 2.01% |
| *LMNA* | 19, 29 | 3 | 1.51% |
| *EXT1* | 19, 30 | 3 | 1.51% |
| *SMARCB1* | 19 | 1 | 0.50% |
| *SMARCE1* | 19 | 1 | 0.50% |
| *TRPS1* | 19 | 1 | 0.50% |
| *FGFR2* | 20, 34, 40 | 12 | 6.03% |
| *CUL7* | 21 | 5 | 2.51% |
| *PCNT* | 21 | 1 | 0.50% |
| *FAM111A* | 21 | 1 | 0.50% |
| *POLE* | 21 | 1 | 0.50% |
| *TBCE* | 21 | 1 | 0.50% |
| *IDS* | 22 | 8 | 4.02% |
| *GALNS* | 22 | 5 | 2.51% |
| *GNPTAB* | 22 | 3 | 1.51% |
| *IDUA* | 22 | 2 | 1.01% |
| *NAGLU* | 22 | 1 | 0.50% |
| *GLB1* | 22 | 1 | 0.50% |
| *EBP* | 23 | 1 | 0.50% |
| *PEX7* | 23 | 1 | 0.50% |
| *COL1A1* | 25, 26 | 6 | 3.02% |
| *ANKH* | 25, 27 | 1 | 0.50% |
| *COL1A2* | 26 | 2 | 1.01% |
| *POLR3A* | 26 | 1 | 0.50% |
| *PHEX* | 27 | 12 | 6.03% |
| *ALPL* | 27 | 1 | 0.50% |
| *CYP27B1* | 27 | 2 | 1.01% |
| *CLCN5* | 27 | 1 | 0.50% |
| *CASR* | 27 | 1 | 0.50% |
| *NF1* | 30 | 5 | 2.51% |
| *FGFR1* | 30, 34, 39 | 2 | 1.01% |
| *PTPN11* | 30 | 2 | 1.01% |
| *ACVR1* | 30 | 1 | 0.50% |
| *KRAS* | 30? | 1 | 0.50% |
| *PIK3CA* | 31 | 3 | 1.51% |
| *FBN2* | 31 | 2 | 1.01% |
| *NSD1* | 31 | 2 | 1.01% |
| *DNMT3A* | 31 | 1 | 0.50% |
| *TGFBR2* | 31 | 1 | 0.50% |
| *SETD2* | 31 | 1 | 0.50% |
| *EZH2* | 31 | 1 | 0.50% |
| *UPF3B* | 31? | 1 | 0.50% |
| *CCN6* | 32 | 2 | 1.01% |
| *SKI* | 34 | 1 | 0.50% |
| *POR* | 34 | 1 | 0.50% |
| *BCL11B* | 34 | 1 | 0.50% |
| *ZIC1* | 34 | 1 | 0.50% |
| *EFNB1* | 35 | 1 | 0.50% |
| *MESP2* | 36 | 1 | 0.50% |
| *NIPBL* | 38 | 4 | 2.01% |
| *SMC1A* | 38 | 1 | 0.50% |
| *RAD21* | 38 | 1 | 0.50% |
| *PORCN* | 39 | 1 | 0.50% |
