# Supplementary material for: Clinical and genetic profiles of postnatal patients with skeletal dysplasia in Guangxi during 8 years: a single-center experience
Source: Orphanet J Rare Dis. 2026 Aug 31;21:305. doi: 10.1186/s13023-026-04414-2 (PMC13528159; doi:10.1186/s13023-026-04414-2)
Supplement: Supplementary file 4 [file 13023_2026_4414_MOESM6_ESM.docx]

Table S4. The number of patients with causative genes not listed in the Nosology of genetic skeletal disorders: 2023 revision.

| **Gene** | **Disorder** | **Disorder type** | **Number of cases** | **Proportion** |
| --- | --- | --- | --- | --- |
| *PIEZO2* | Arthrogryposis | Neurodevelopmental disorder including skeletal abnormalities | 2 | 3.03% |
| *ATRX* | Intellectual disability-hypotonic facies syndrome | Neurodevelopmental disorder including skeletal abnormalities | 2 | 3.03% |
| *CASK* | Intellectual developmental disorder | Neurodevelopmental disorder including skeletal abnormalities | 2 | 3.03% |
| *DDX3X* | Intellectual developmental disorder | Neurodevelopmental disorder including skeletal abnormalities | 2 | 3.03% |
| *KMT2A* | Wiedemann-Steiner syndrome | Neurodevelopmental disorder including skeletal abnormalities | 2 | 3.03% |
| *SETBP1* | Schinzel-Giedion midface retraction syndrome | Neurodevelopmental disorder including skeletal abnormalities | 1 | 1.52% |
| *USP9X* | Intellectual developmental disorder | Neurodevelopmental disorder including skeletal abnormalities | 1 | 1.52% |
| *IGHMBP2* | Neuronopathy, distal hereditary motor | Neurodevelopmental disorder including skeletal abnormalities | 1 | 1.52% |
| *SCN1A* | Developmental and epileptic encephalopathy 6B | Neurodevelopmental disorder including skeletal abnormalities | 1 | 1.52% |
| *MECP2* | Rett syndrome | Neurodevelopmental disorder including skeletal abnormalities | 1 | 1.52% |
| *SMARCC2* | Coffin-Siris syndrome 8 | Neurodevelopmental disorder including skeletal abnormalities | 1 | 1.52% |
| *KANSL1* | Koolen-De Vries syndrome | Neurodevelopmental disorder including skeletal abnormalities | 1 | 1.52% |
| *SRCAP* | Floating-Harbor syndrome | Neurodevelopmental disorder including skeletal abnormalities | 1 | 1.52% |
| *NFASC* | Neurodevelopmental disorder with central and peripheral motor dysfunction | Neurodevelopmental disorder including skeletal abnormalities | 1 | 1.52% |
| *MAGEL2* | Schaaf-Yang syndrome | Neurodevelopmental disorder including skeletal abnormalities | 1 | 1.52% |
| *MAF* | Ayme-Gripp syndrome | Neurodevelopmental disorder including skeletal abnormalities | 1 | 1.52% |
| *MOCS1* | Molybdenum cofactor deficiency A | Neurodevelopmental disorder including skeletal abnormalities | 1 | 1.52% |
| *FBXO11* | Intellectual developmental disorder | Neurodevelopmental disorder including skeletal abnormalities | 1 | 1.52% |
| *ASXL3* | Bainbridge-Ropers syndrome | Neurodevelopmental disorder including skeletal abnormalities | 1 | 1.52% |
| *COL6A3* | Ullrich congenital muscular dystrophy 1 | Muscular disorder including skeletal abnormalities | 2 | 3.03% |
| *DMD* | Duchenne muscular dystrophy | Muscular disorder including skeletal abnormalities | 2 | 3.03% |
| *DYNC1H1* | Charcot-Marie-Tooth disease | Muscular disorder including skeletal abnormalities | 1 | 1.52% |
| *ASCC1* | Spinal muscular atrophy with congenital bone fractures 2 | Muscular disorder including skeletal abnormalities | 1 | 1.52% |
| *KLHL40* | Nemaline myopathy 8 | Muscular disorder including skeletal abnormalities | 1 | 1.52% |
| *KMT2D* | Kabuki syndrome 1 | Multisystemic disorder including skeletal abnormalities | 2 | 3.03% |
| *OCRL* | Lowe syndrome | Multisystemic disorder including skeletal abnormalities | 2 | 3.03% |
| *SHOC2* | Noonan syndrome-like with loose anagen hair 1 | Multisystemic disorder including skeletal abnormalities | 2 | 3.03% |
| *COL3A1* | Ehlers-Danlos syndrome | Multisystemic disorder including skeletal abnormalities | 1 | 1.52% |
| *CPLANE1* | Joubert syndrome 17; Orofaciodigital syndrome VI | Multisystemic disorder including skeletal abnormalities | 1 | 1.52% |
| *RPS6KA3* | Coffin-Lowry syndrome | Multisystemic disorder including skeletal abnormalities | 1 | 1.52% |
| *TRIM37* | Mulibrey nanism | Multisystemic disorder including skeletal abnormalities | 1 | 1.52% |
| *BBS2* | Bardet-Biedl syndrome 2 | Multisystemic disorder including skeletal abnormalities | 1 | 1.52% |
| *ERCC2* | Cerebrooculofacioskeletal syndrome 2 | Multisystemic disorder including skeletal abnormalities | 1 | 1.52% |
| *CHD7* | CHARGE syndrome | Multisystemic disorder including skeletal abnormalities | 1 | 1.52% |
| *PTCH1* | Basal cell nevus syndrome 1 | Multisystemic disorder including skeletal abnormalities | 1 | 1.52% |
| *CCDC22* | Ritscher-Schinzel syndrome 2 | Multisystemic disorder including skeletal abnormalities | 1 | 1.52% |
| *ROBO3* | Gaze palsy, familial horizontal | Multisystemic disorder including skeletal abnormalities | 1 | 1.52% |
| *PITX2* | Axenfeld-Rieger syndrome, type 1 | Multisystemic disorder including skeletal abnormalities | 1 | 1.52% |
| *TNXB* | Ehlers-Danlos syndrome | Multisystemic disorder including skeletal abnormalities | 1 | 1.52% |
| *ANKRD11* | KBG syndrome | Multisystemic disorder including skeletal abnormalities | 1 | 1.52% |
| *TNNI2* | Arthrogryposis, distal | Skeletal abnormalities | 1 | 1.52% |
| *WAS* | Wiskott-Aldrich syndrome | Hemopathy including skeletal abnormalities | 1 | 1.52% |
| *NKX2-1* | Choreoathetosis, hypothyroidism, and neonatal respiratory distress | Neurodevelopmental disorder | 1 | 1.52% |
| *CC2D1A* | Intellectual developmental disorder, autosomal recessive 3 | Neurodevelopmental disorder | 1 | 1.52% |
| *HNRNPU* | Developmental and epileptic encephalopathy 54 | Neurodevelopmental disorder | 1 | 1.52% |
| *OPHN1* | Intellectual developmental disorder | Neurodevelopmental disorder | 1 | 1.52% |
| *SMN1* | Spinal muscular atrophy-1 | Muscular disorder | 1 | 1.52% |
| *GATA3* | Hypoparathyroidism, sensorineural deafness, and renal dysplasia | Multisystemic disorder | 1 | 1.52% |
| *ATM* | Ataxia-telangiectasia | Multisystemic disorder | 1 | 1.52% |
| *SAMD9L* | Ataxia-pancytopenia syndrome | Multisystemic disorder | 1 | 1.52% |
| *GFM1* | Combined oxidative phosphorylation deficiency 1 | Multisystemic disorder | 1 | 1.52% |
| *DUOX2* | Thyroid dyshormonogenesis 6 | Metabolic disease | 1 | 1.52% |
| *EPAS1* | Erythrocytosis | Hemopathy | 1 | 1.52% |
| *SPTB* | Spherocytosis | Hemopathy | 1 | 1.52% |
| *SRD5A2* | Pseudovaginal perineoscrotal hypospadias | Gonadal dysplasia | 1 | 1.52% |
| *GJB2; GJB6* | Deafness | Deafness | 1 | 1.52% |

*SRD5A2* and *GJB2/GJB6* variants were identified in patients with dual diagnosis.
