# Supplementary material for: Clinical and genetic profiles of postnatal patients with skeletal dysplasia in Guangxi during 8 years: a single-center experience
Source: Orphanet J Rare Dis. 2026 Aug 31;21:305. doi: 10.1186/s13023-026-04414-2 (PMC13528159; doi:10.1186/s13023-026-04414-2)
Supplement: Supplementary file 5 [file 13023_2026_4414_MOESM7_ESM.docx]

Table S5 The number of patients harbored pathogenic variants but without SD.

| Gene | Patients without SD | Patients  with SD | Gene | Patients without SD | Patients with SD |
| --- | --- | --- | --- | --- | --- |
| NF1 | 19 | 4 | NOTCH2 | 1 | 0 |
| PTPN11 | 6 | 2 | SMARCE1 | 1 | 1 |
| ARID1B | 5 | 0 | SHH | 1 | 0 |
| NIPBL | 5 | 6 | ABCC9 | 1 | 0 |
| LMNA | 5 | 3 | CC2D2A | 1 | 0 |
| FGFR1 | 3 | 2 | ACTG1 | 1 | 0 |
| MAP2K1 | 3 | 0 | CUL7 | 1 | 7 |
| TCOF1 | 3 | 0 | NFIX | 1 | 0 |
| FGFR2 | 2 | 16 | SMC3 | 1 | 0 |
| TMEM67 | 2 | 0 | FGFR3 | 1 | 92 |
| SMC1A | 2 | 1 | PGM3 | 1 | 0 |
| EP300 | 2 | 0 | COL11A1 | 1 | 1 |
| CREBBP | 2 | 4 | TFAP2B | 1 | 0 |
| COL1A1 | 2 | 21 | TP63 | 1 | 3 |
| NSD1 | 2 | 2 | NOTCH1 | 1 | 0 |
| GLB1 | 2 | 1 | EFNB1 | 1 | 1 |
| CLCN5 | 2 | 1 | SBDS | 1 | 0 |
| MYCN | 2 | 0 | ARSE | 1 | 0 |
| FLNA | 1 | 0 | PIK3CA | 1 | 3 |
| PRKAR1A | 1 | 3 | ASXL1 | 1 | 0 |
| OFD1 | 1 | 0 | MID1 | 1 | 0 |
| SPECC1L | 1 | 0 | OBSL1 | 1 | 0 |
| HRAS | 1 | 0 | MYH3 | 1 | 2 |
| HDAC8 | 1 | 0 | KCNJ2 | 1 | 0 |
| GUSB | 1 | 0 |  |  |  |

SD, skeletal dysplasia. The genes were all listed in the Nosology of genetic skeletal disorders: 2023 Revision. One patient with SD harbors two *NF1* mutations.
