# Supplementary material for: Clinical and genetic profiles of postnatal patients with skeletal dysplasia in Guangxi during 8 years: a single-center experience
Source: Orphanet J Rare Dis. 2026 Aug 31;21:305. doi: 10.1186/s13023-026-04414-2 (PMC13528159; doi:10.1186/s13023-026-04414-2)
Supplement: Supplementary file 6 [file 13023_2026_4414_MOESM8_ESM.docx]

Table S6. Diagnostic yield of skeletal dysplasia from NGS strategies in different cohorts in key publications

| **Author** | **Journal** | **Sample** | **Strategy** | **Sequenced** | **Diagnostic yield** |
| --- | --- | --- | --- | --- | --- |
| This study | This journal | Postnatal | WES | 388 | 65% |
|  |  |  | SS | 46 | 80% |
| Najjar et al., 2025 | Front Endocrinol | Pre- and post-natal | WES, WGS | 262 | 50% |
|  |  |  | SS | 208 | 18% |
| Jacob et al., 2025 | Eur J Hum Genet | Pre- and post-natal | WES, WGS | 152 | 71% |
|  |  |  | SS | 50 | 74% |
| Li et al., 2023 | Chin Med J | Pre- and post-natal | WES, WGS, SS, MLPA | 985 | 59% |
| Scocchia et al., 2021 | Orphanet J Rare Dis | Pre- and post-natal | TES | 543 | 42% |
| Lv et al., 2021 | Front Cell Dev Biol | Pre- and post-natal | TES | 223 | 49% |
| Silveira et al., 2021 | Am J Med Genet C | Postnatal | TES | 114 | 77% |
| Maddirevula et al., 2018 | Genet Med | Postnatal | TES, WES | 288 | 44% |
| Bae et al., 2016 | Genet Med | Postnatal | TES, SS | 185 | 52% |
| Retterer et al., 2016 | Genet Med | Postnatal | WES | 139 | 39% |
| Zhang et al., 2015 | J Hum Genet. | Postnatal | TES | 82 | 54% |

WES, whole-exome sequencing; WGS, whole-genome sequencing; SS, Sanger sequencing; MLPA, multiplex ligation-dependent probe amplification; TES, targeted exome sequencing.
